# Ketamine-Induced Cholangiopathy With Concomitant Hemorrhagic Cystitis: An Emerging and Underrecognized Cause of Cholestasis

**DOI:** 10.7759/cureus.99972

**Published:** 2025-12-23

**Authors:** Kamran Nazir, Waqas Ahmed, Salman Rafi, Adeel Ahmad, Asim Khaleeq

**Affiliations:** 1 Medicine, Lancashire Teaching Hospitals NHS Foundation Trust, Chorley, GBR; 2 Respiratory Medicine, Lancashire Teaching Hospitals NHS Foundation Trust, Chorley, GBR; 3 Cardiology, Lancashire Teaching Hospitals NHS Foundation Trust, Chorley, GBR; 4 Internal Medicine, Kettering General Hospital NHS Foundation Trust, Kettering, GBR

**Keywords:** biliary stricture, hepatobiliary complication, ketamine-induced cholangiopathy, ketamine-induced cystitis, recurrent hemorrhagic cystitis

## Abstract

Ketamine-induced cholangiopathy (KIC) is a rare but increasingly recognized manifestation of chronic ketamine toxicity, often accompanied by urinary tract injury such as hemorrhagic cystitis. We report the case of a 55-year-old female with recurrent hemorrhagic cystitis and a long-standing history of intermittent recreational ketamine use who presented with asymptomatic cholestatic liver enzyme abnormalities. Hepatobiliary ultrasound revealed hepatic steatosis without ductal dilatation, and magnetic resonance cholangiopancreatography demonstrated multifocal biliary stricturing with a beaded appearance of the intrahepatic bile ducts. Serological testing for autoimmune and infectious etiologies was negative. Cystoscopy revealed inflammatory bladder changes, and histopathology confirmed ketamine-induced hemorrhagic cystitis. Following cessation of ketamine use, the patient demonstrated biochemical improvement. This case emphasizes the multisystem toxic potential of ketamine, highlighting the importance of recognizing KIC with concomitant hemorrhagic cystitis as an emerging cause of cholestasis and urinary tract injury.

## Introduction

Ketamine, synthesized in the early 1960s, is a phencyclidine derivative that functions as a dissociative anesthetic through potent antagonism of N-methyl-D-aspartate (NMDA) receptors [1]. Clinically, it has been widely utilized for anesthesia and analgesia owing to its rapid onset, profound pain control, and hemodynamic stability. Ketamine undergoes extensive hepatic metabolism via cytochrome P450 enzymes, predominantly CYP2B6 and CYP3A4, to form nor-ketamine, which is excreted mainly through the kidneys (≈90%) and, to a lesser extent, via bile (≈10%) [2]. These pharmacologic properties make ketamine highly effective in clinical practice but also create pathways for toxicity when used in excess.

Despite its therapeutic value, increasing recreational use has revealed significant hepatobiliary and urinary complications [3,4]. This trend is clinically important, as many affected patients present first to general medical or emergency settings. The precise pathogenesis of ketamine-induced liver injury remains uncertain, though proposed mechanisms include NMDA receptor-mediated smooth muscle relaxation of bile ducts leading to ductal dilatation and cholestasis [5], direct epithelial toxicity from ketamine metabolites [6], and vagally mediated reductions in gallbladder motility causing bile stasis [7]. Together, these mechanisms may contribute to a progressive cholangiopathy in chronic users.

Chronic ketamine abuse, typically over a median duration of 24 months [8], has been associated with cholangiopathy resembling primary sclerosing cholangitis (PSC). Acute ketamine exposure may lead to drug-induced liver injury [9], whereas prolonged or heavy use more often results in cholangiopathy [10,11], manifesting with abdominal pain, jaundice, and cholestatic liver enzyme abnormalities [12]. Magnetic resonance cholangiopancreatography (MRCP) often demonstrates multifocal intrahepatic and extrahepatic ductal strictures and dilatations, consistent with ketamine-induced cholangiopathy (KIC) [9].

This case highlights an increasingly recognized but still underdiagnosed complication of recreational ketamine use, underscoring the need for clinicians to consider ketamine-associated cholangiopathy in patients presenting with unexplained cholestasis.

## Case presentation

A 55-year-old physically active and independent woman (Performance Status 1), with no history of smoking and only occasional social alcohol consumption, was referred by her general practitioner in January 2025 for evaluation of persistently elevated cholestatic liver function tests (LFTs) over the preceding six months. Her past medical history was notable for well-controlled hypertension and recurrent episodes of hemorrhagic cystitis, for which she was under ongoing urological follow-up.

On clinical assessment, she appeared well and remained entirely asymptomatic. A comprehensive systemic review was unremarkable, and both abdominal and hepatic examinations revealed no abnormalities. The primary objective was to identify the underlying cause of the persistently deranged cholestatic LFTs.

Initial biochemical investigations demonstrated chronically elevated alkaline phosphatase (ALP 750 U/L) and gamma-glutamyl transferase (GGT 1250 U/L), which had persisted for more than six months without diagnostic clarification. The detailed LFT profile is presented in Table 1. Noninvasive liver screening, including viral (HAV, HBV, HCV, HEV, and HIV), autoimmune (antinuclear antibodies (ANA), antineutrophil cytoplasmic antibodies (ANCA), and IgG4), and metabolic (iron studies, ceruloplasmin, and alpha-1 antitrypsin) panels, yielded no abnormalities (Table 2). Abdominal ultrasonography was unremarkable except for mild hepatic steatosis. Further evaluation to exclude secondary causes of sclerosing cholangitis, including HIV serology, IgG4 levels, autoimmune markers, and tumor markers (CA 19-9), demonstrated no significant findings (Table 3).

**Table 1:** LFTs Markedly elevated ALP and GGT suggest a cholestatic pattern, likely due to an obstructive biliary cause such as a stone, stricture, or tumor. ALP, alkaline phosphatase; ALT, alanine aminotransferase; GGT, gamma-glutamyl transferase; LFTs, liver function tests

LFTs	Patient’s results	Reference range
Bilirubin	<5	3-21 µmol/L
ALP	673	40-129 IU/L
GGT	1295	10-60 IU/L
ALT	171	10-45 IU/L
Albumin	45	35-50 g/L

**Table 2:** Noninvasive liver screening Unremarkable noninvasive liver screening excludes viral hepatitis, autoimmune hepatitis, hereditary hemochromatosis, Wilson disease, alpha-1 antitrypsin deficiency, or primary biliary cholangitis. Anti-HAV Ab, anti-hepatitis A virus antibody; Anti-HCV Ab, anti-hepatitis C virus antibody; Anti-HEV Ab, anti-hepatitis E virus antibody; HBsAg, hepatitis B surface antigen; S/CO, signal-to-cutoff ratio

Noninvasive liver screening	Patient’s results	Reference ranges
Anti-HAV Ab	Negative	Negative (S/CO < assay cutoff)
HBsAg	Negative	Negative (S/CO < assay cutoff)
Anti-HCV Ab	Negative	Negative (S/CO < assay cutoff)
Anti-HEV Ab	Negative	Negative (S/CO < assay cutoff)
Autoimmune screening	Negative	Negative (S/CO < assay cutoff)
Serum ferritin	51	15-150 µg/L
Iron saturation	24	20-50%
IgA	2.1	0.7-4.0 g/L
IgM	0.8	0.4-2.3 g/L
IgG	11.9	7-16 g/L
Alpha-1 antitrypsin level	1.5	1.0-2.0 g/L

**Table 3:** Primary and secondary sclerosing cholangitis screening tests Unremarkable PSC (negative ANA, ANCA, and no evidence of IBD) and negative secondary sclerosing cholangitis, including infections (HIV and Ascaris), autoimmune cholangitis (negative IgG4), or sinister pathology such as cholangiocarcinoma. ANA: Negative - no autoimmune connective tissue disease; Positive - may indicate autoimmune disorders depending on titer and pattern. ANCA: Negative - no ANCA-associated vasculitis; Positive - may suggest vasculitic disease depending on ANCA type and titer. ANA, antinuclear antibodies; ANCA, antineutrophil cytoplasmic antibodies; CA 19-9, carbohydrate antigen 19-9; IBD, inflammatory bowel disease; PSC, primary sclerosing cholangitis; S/CO, signal-to-cutoff ratio

Primary and secondary sclerosing cholangitis screening	Patient’s results	Reference range
HIV serology	Nonreactive	Reactive/nonreactive (S/CO ratio)
IgG4 antibodies	1.26	0.03-2.01 g/L
ANA	Negative	Negative
ANCA	Negative	Negative
CA 19-9	13	0-37 U/mL

Fibrosis assessment showed a FIB-4 score of 1.4, and transient elastography (FibroScan; Table 4) revealed a liver stiffness measurement (LSM) of 6.4 kPa and a controlled attenuation parameter of 310 dB/m, consistent with mild hepatic steatosis and no significant fibrosis.

**Table 4:** FibroScan LSM <7 kPa is consistent with F0-F1 fibrosis, indicating mild fibrosis and effectively ruling out advanced fibrosis or cirrhosis. AUROC, area under the receiver operating characteristic; CAP, controlled attenuation parameter; LSM, liver stiffness measurement

FibroScan parameters	Patient’s results	Units	Reference range
LSM	6.4	kPa	<7 kPa: F0/F1 fibrosis; 7-8 kPa: F2 fibrosis; 10-12 kPa: F3 fibrosis; >12 kPa: cACLD (F3/F4); >14 kPa: cirrhosis (F4)
CAP	310	dB/m	<238: normal; 238-260: mild steatosis; 260-290: moderate steatosis; >290: severe steatosis
AUROC	0.81	0-1	0.8-0.99

**Figure 1:**
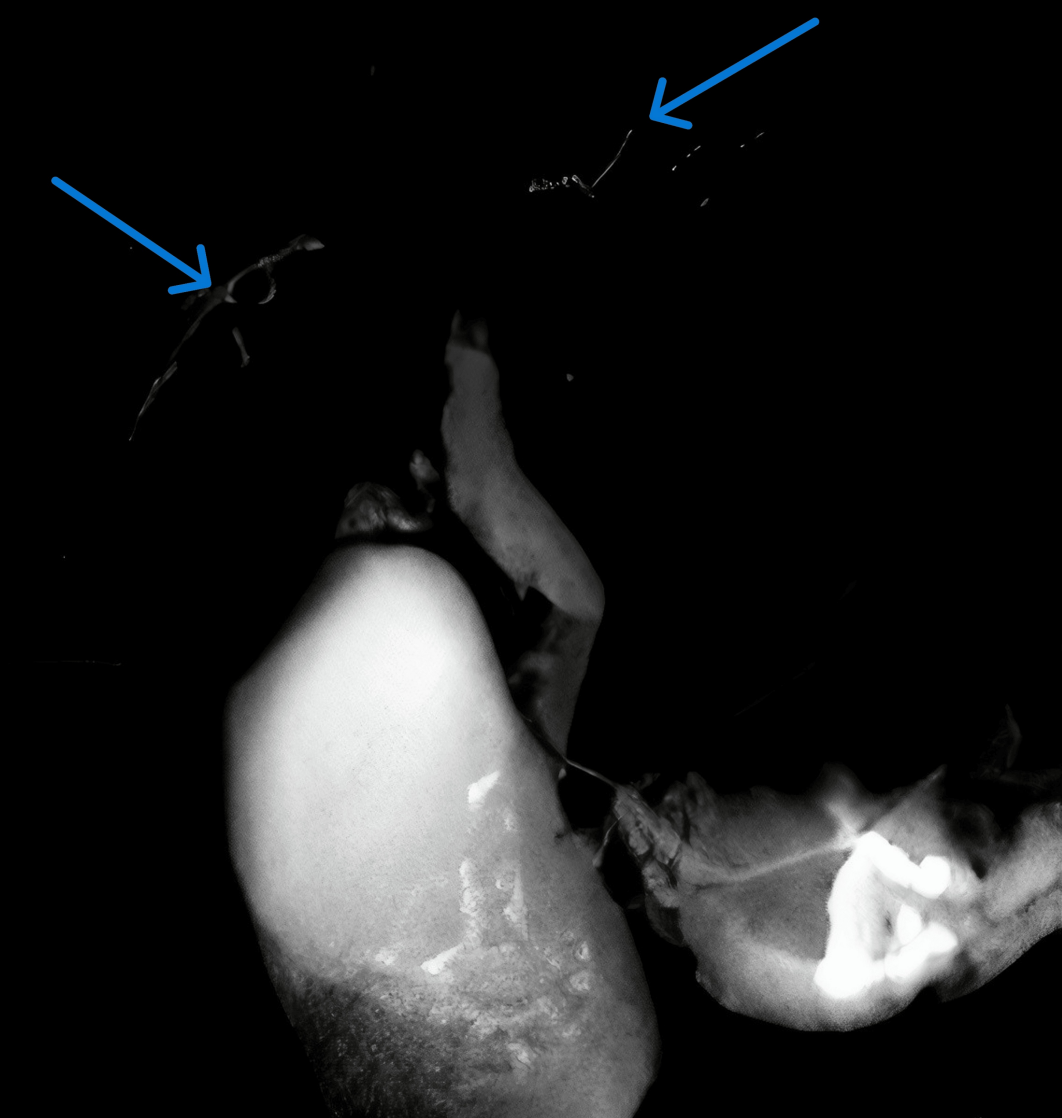
MRCP image demonstrating multifocal intrahepatic biliary strictures with a classic “beaded” appearance (arrows) These alternating narrowings and dilatations are characteristic of cholangiopathy and raise suspicion for ketamine-related biliary injury. MRCP, magnetic resonance cholangiopancreatography

**Figure 2:**
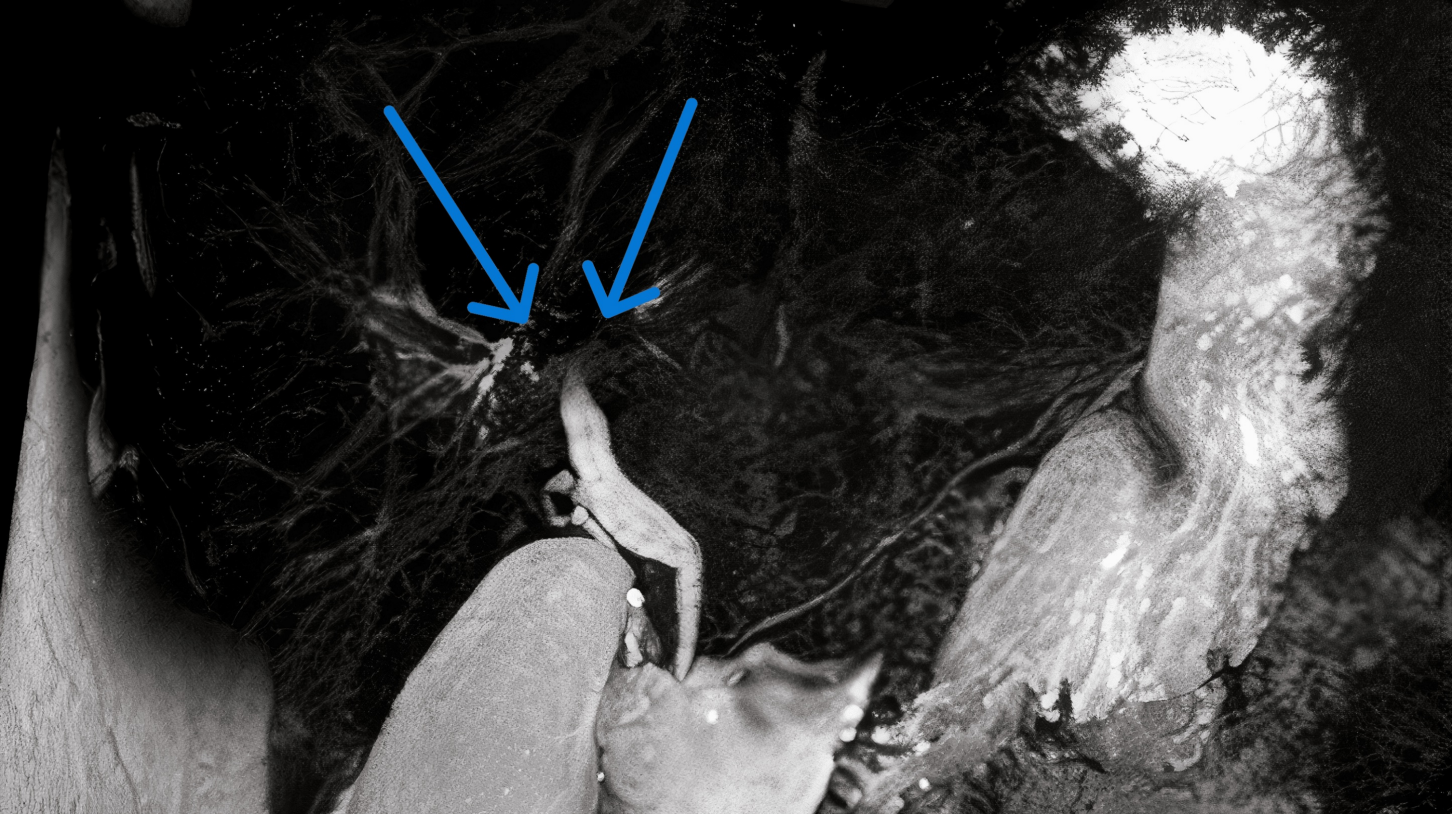
MRCP image highlighting pronounced central biliary stenosis (arrows), predominantly affecting the proximal intrahepatic ducts The smooth tapering pattern helps differentiate drug-induced cholangiopathy from inflammatory causes such as PSC. MRCP, magnetic resonance cholangiopancreatography; PSC, primary sclerosing cholangitis

**Figure 3:**
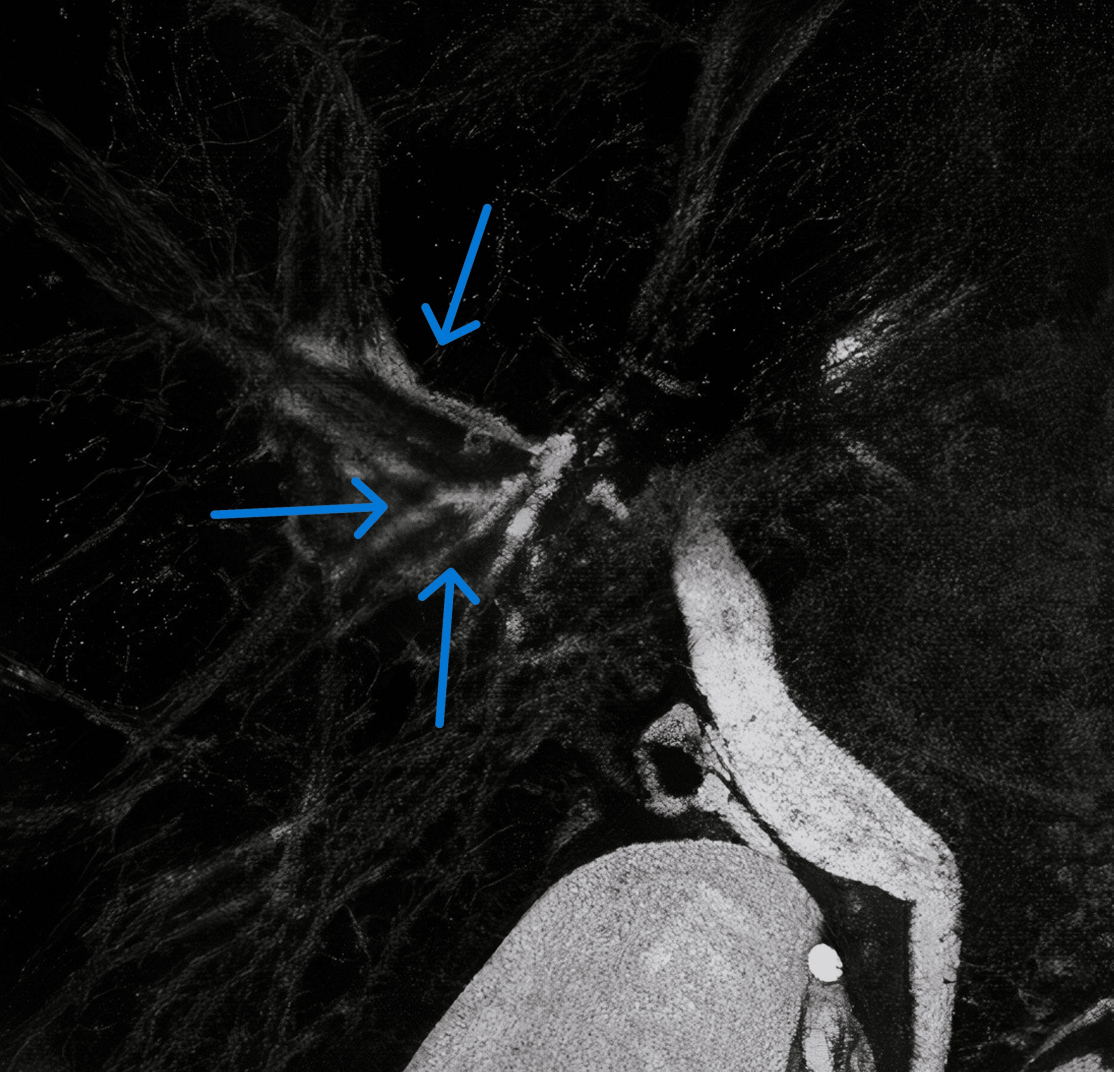
MRCP image showing segmental stricturing of the intrahepatic bile ducts (arrows), with focal areas of narrowing interspersed with normal-caliber ducts These findings support multifocal biliary involvement consistent with KIC. KIC, ketamine-induced cholangiopathy; MRCP, magnetic resonance cholangiopancreatography

**Figure 4:**
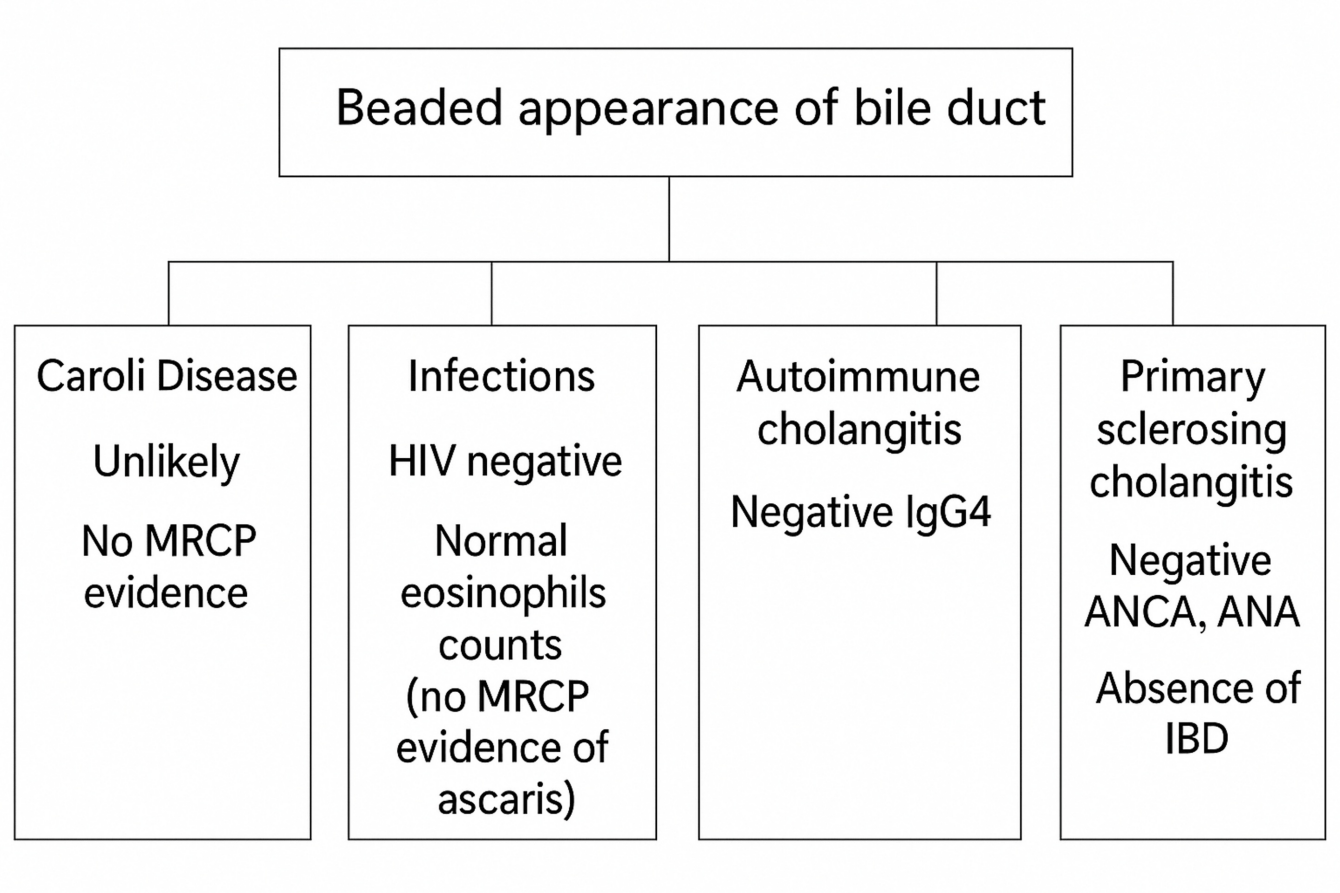
Beaded appearance of bile duct - primary and secondary sclerosing cholangitis screening tests ANCA, antineutrophil cytoplasmic antibodies; ANA, antinuclear antibodies; IBD, inflammatory bowel disease; MRCP, magnetic resonance cholangiopancreatography Image credit: Kamran Nazir

MRCP demonstrated multifocal stricturing and a characteristic “beaded” appearance of the intrahepatic bile ducts, findings suggestive of either PSC or a secondary sclerosing process, notably KIC. Representative MRCP images illustrating these features are shown in Figure 1, Figure 2, and Figure 3. A comprehensive diagnostic workup to rule out other etiologies for secondary sclerosing cholangitis, including HIV serology, ANCA, ANA, IgG4 subclass, and tumor marker CA 19-9, was entirely negative. The systematic approach used to exclude alternative causes of secondary sclerosing cholangitis is summarized in Figure 4.

**Figure 5:**
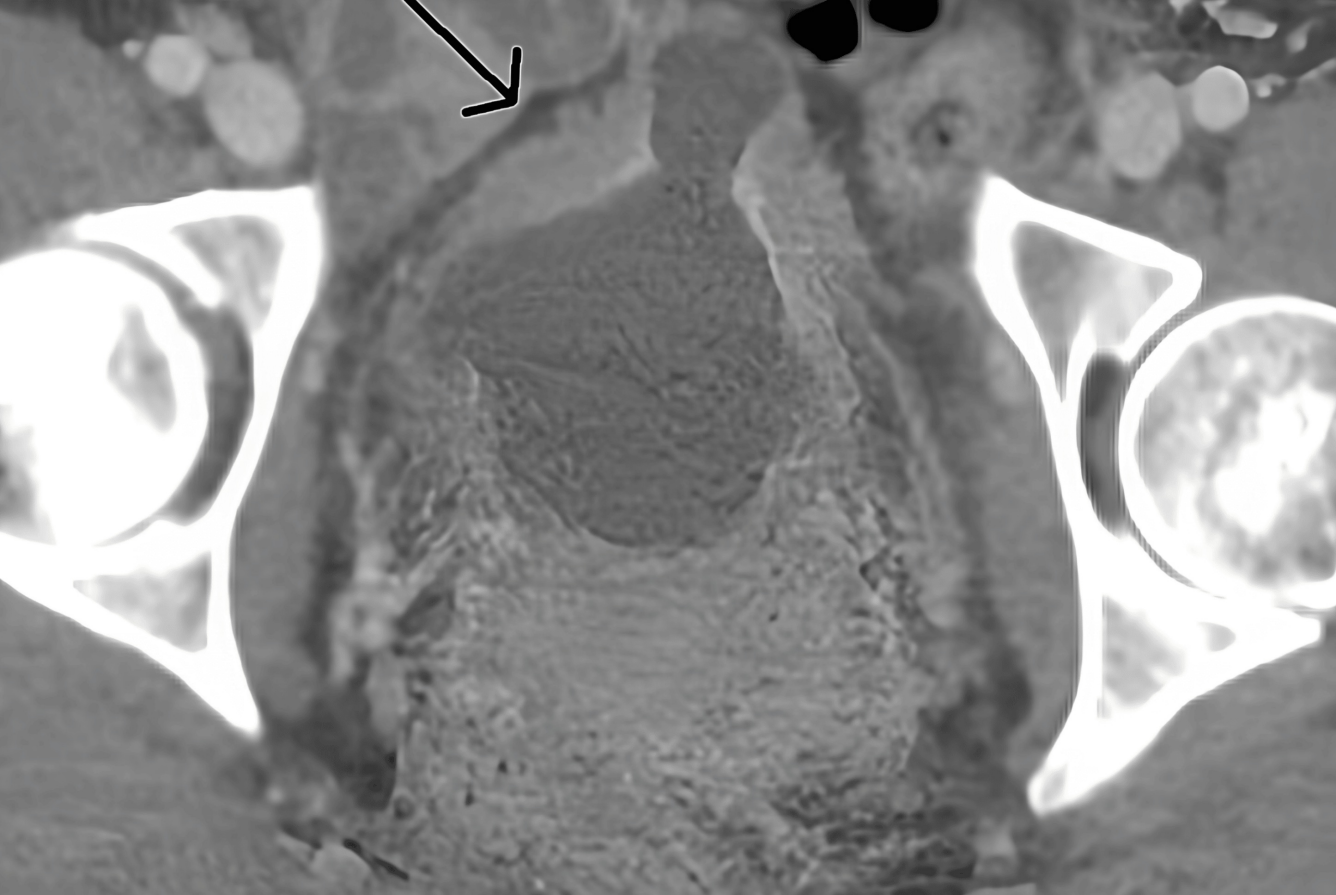
Axial CT scan of the pelvis demonstrating circumferential bladder wall thickening (arrow), a radiological feature suggestive of chronic inflammatory changes associated with ketamine-induced hemorrhagic cystitis

Concurrent urological evaluation for recurrent hemorrhagic cystitis revealed chronic inflammatory changes on cystoscopy and bladder biopsy, consistent with ketamine-associated cystitis. A CT scan of the pelvis demonstrated diffuse bladder wall thickening, as shown in Figure 5. Correlating these findings with her confirmed decade-long history of intermittent ketamine use and the exclusion of alternative hepatobiliary causes established the diagnosis of KIC, a form of secondary sclerosing cholangitis.

The case was discussed at a hepato-pancreato-biliary multidisciplinary team (MDT) meeting, where consensus supported the diagnosis of ketamine-induced secondary sclerosing cholangitis. The MDT recommended strict abstinence from ketamine to prevent further hepatic injury and disease progression, annual follow-up with MRCP to monitor biliary stricturing and hepatic fibrosis, and referral to mental health services for support with abstinence and management of underlying psychosocial factors contributing to ketamine use. The patient was also placed under ongoing multidisciplinary surveillance, integrating hepatology, urology, and psychological care to manage potential hepatobiliary and urological complications and optimize long-term outcomes.

## Discussion

KIC is an increasingly recognized but still underdiagnosed form of secondary sclerosing cholangitis, predominantly reported in individuals with chronic recreational ketamine use [3,10,12]. Consistent with previously published case series and systematic reviews, our patient demonstrated a predominantly cholestatic biochemical profile with markedly elevated ALP and GGT, preserved synthetic liver function, and characteristic MRCP findings of multifocal biliary stricturing with a beaded appearance [9,10,12].

The radiological resemblance between KIC and PSC represents a major diagnostic challenge. Prior studies have shown that KIC often exhibits smoother, less irregular strictures compared to the classic “pruned-tree” appearance seen in advanced PSC [10,12]. Consistent with this, MRCP in our patient demonstrated mild, smooth intrahepatic stricturing without significant ductal irregularity or advanced fibrosis, supporting a drug-induced rather than autoimmune etiology.

In contrast to PSC, which is strongly associated with inflammatory bowel disease in up to 70-80% of cases, our patient had no gastrointestinal symptoms, no history of IBD, and negative autoimmune serology, including ANA and ANCA [10,12]. These findings mirror those reported in ketamine-related cholangiopathy case series, where autoimmune markers are typically absent, and IgG4 levels remain within normal limits [6,10].

An important distinguishing feature in our case was the coexistence of ketamine-induced hemorrhagic cystitis. Previous reports highlight that urinary tract involvement often precedes hepatobiliary manifestations and may serve as a key diagnostic clue to systemic ketamine toxicity [3,6,12]. The presence of chronic cystitis with confirmatory cystoscopic and histological findings strongly supports ketamine exposure as the unifying pathogenic mechanism, a feature absent in PSC.

The pathophysiology of KIC remains incompletely understood. Proposed mechanisms include direct biliary epithelial toxicity from ketamine metabolites excreted in bile, NMDA receptor-mediated smooth muscle dysfunction leading to bile stasis, and ischemic injury related to vasoconstriction [5-7]. Variability in disease progression reported in the literature may reflect differences in duration, dose, and patterns of ketamine use, as well as delays in diagnosis and cessation [12].

Importantly, several studies have demonstrated partial or complete biochemical improvement following ketamine abstinence, particularly when cessation occurs early in the disease course [6,12]. Our patient similarly showed significant biochemical recovery following strict abstinence, reinforcing published evidence that ketamine cessation remains the cornerstone of management and may prevent progression to irreversible biliary fibrosis or cirrhosis.

## Conclusions

KIC is an emerging cause of secondary sclerosing cholangitis that closely mimics PSC. A thorough drug history, exclusion of autoimmune and infectious causes, and recognition of associated ketamine-induced cystitis are critical for diagnosis. Early identification and strict ketamine cessation are essential, as timely withdrawal may lead to biochemical improvement and prevent progression to irreversible biliary fibrosis.
